# Potential Stable Low-Permeation Rate Standard Based on Micro-machined Silicon

**DOI:** 10.6028/jres.120.021

**Published:** 2015-12-15

**Authors:** Adrian Verwolf, Chris Poling, Nick Barbosa, Grady White, Nikki Rentz

**Affiliations:** 1 Focused Labs, LLC, Denver, CO 80239; 2 National Institute of Standards and Technology, Boulder, CO 80305

**Keywords:** calibration, chilled mirror hygrometer, mass loss, permeation, Si, standard

## Abstract

Silicon wafers with micro-machined holes were evaluated for use as low-permeation-rate standard artifacts. Accuracy, stability, and reliability were assessed. Two independent experimental techniques for evaluating permeation were used: chilled mirror hygrometer and mass loss. The wafers exhibited a well-defined linear relationship between hole area and resultant water partial pressure for both techniques, although the mass loss curve exhibited a constant vertical offset from the hygrometer curve, attributed to water loss through the O-ring seal. In contrast to polymer permeation standards, Si wafers provided long-term reproducible permeation rates. However, they were also highly fragile, with most of them cracking during the course of the investigation.

## 1. Introduction

There is broad industry need for a standard reference material (SRM) to provide a means to calibrate vapor permeation test systems. Such applications exist in the food packaging industry, pharmaceutical companies, polymer film manufacturers, and analytical chemistry. Applications that require such standards include polymeric barrier encapsulation of water-sensitive devices, e.g., active implantable medical components, avionics systems, and organic electronics. All of these industries have stated a need for more reliable water permeation data, particularly at trace levels, traceable to reliable standards. The lack of permeation standards is also an impediment to research efforts. Reliable prediction of water infiltration into coated electronic assemblies is dependent on accurate experimental measurement of water transport through candidate polymers. ASTM standard test methods [1–4] are currently used by industry to measure permeation of water vapor and organic compounds through polymer films. The instruments and test fixtures that are used come principally from four manufacturers; however, the vendors’ recommended calibration technique provides no assurance of accuracy and is dependent on the presumed long-term stability of the polymer check membranes. Notably, ASTM recently stopped selling its own reference membrane standard for organic permeation due to concerns about the material’s degradation over time. For these reasons, there is a widespread desire for a validated, highly stable, non-polymeric reference standard for calibrating permeation test instruments.

This work addresses the possible use of micro-machined silicon wafers as low-rate water vapor permeation standards, addressing permeation rate as a function of hole area, reproducibility, and stability over time. The approach used for this work involved both water vapor/frost point measurements and mass loss measurements. Because the mass of the permeation test cell designed for this work could be measured with a sensitive analytical balance, online water vapor frost-point measurements were directly compared to periodic measurements of mass loss resulting from molecular transport. This approach gave two independent paths to measure permeability, thereby providing a self-consistency check.

## 2. Experimental Procedure

### 2.1 Specimen Preparation

#### 2.1.1 Material Choice

The choice of material for this work was driven by three constraints. Because the long-term storage conditions would be controlled by the end user and, therefore, outside of the control of the manufacturer, the permeation artifact had to be temporally stable to visible and UV light and to a fairly wide range of temperatures (e.g., 0 °C to 85 °C). Finally, because water is highly corrosive, the material needed to be chemically inert to water.

Based upon these criteria, three candidate materials were initially considered: stainless steel (SS) foil, aluminum oxide (Al_2_O_3_) wafers, and silicon (Si) wafers. SS met the requirements of being stable over a wide temperature range and under exposure to visible and UV radiation. In addition, it is a very tough material and resists fracture and tearing. However, SS foils are very malleable, raising concerns about whether small wrinkles in the material would allow water vapor a path around the SS barrier, i.e., whether the material would seal adequately to prevent water flow on the hundreds of parts per million level and whether precision holes in the foil might distort with usage over time. Al_2_O_3_ had the advantages of being chemically inert, stable, and strong. However, facilities for manufacturing the small, well-defined holes needed for this work are not readily available. In addition, Al_2_O_3_ experiences slow crack growth in the presence of water (both liquid and vapor) and stress. This behavior made the suitability of Al_2_O_3_ as a membrane material for water permeation questionable. The last material considered was Si. Like the two other materials, Si is stable over a wide temperature range and under exposure to visible and UV radiation. In addition, standard industry techniques exist to micro-machine small holes in Si precisely. Finally, unlike SS, the brittle nature of Si means that it will fracture under stress, rather than deform to accommodate the stress, resulting in a discrete change in permeability that would be easily recognized. Therefore, despite its tendency toward brittle failure, the physical and chemical stability of Si, coupled with the well-established micro-machining techniques available for fabrication of small holes, led us to choose Si as our permeation standard candidate material.

#### 2.1.2 Si Wafer Fabrication

**Fig. 1:**
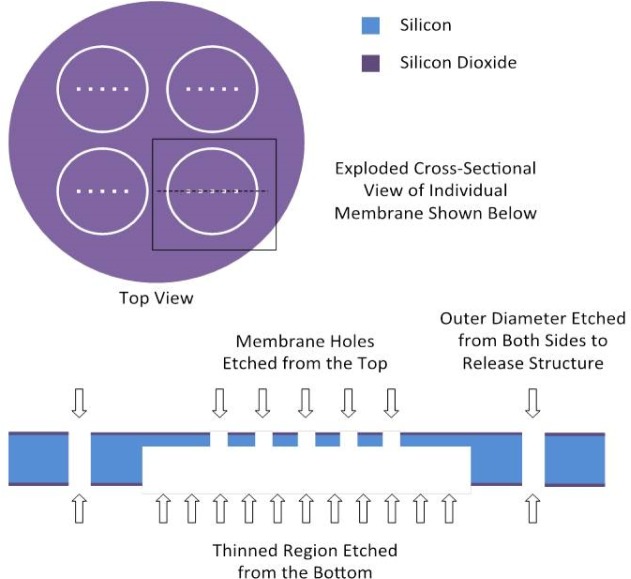
Schematic showing the layout and processing steps used to fabricate silicon permeation membranes. Four membranes were fabricated per wafer with a choice of hole patterns available for individual membranes.

Permeation membranes were fabricated from 7.62 cm diameter (i.e., standard industry three-inch diameter), double-side-polished, silicon wafers using conventional cleanroom techniques. Four, 2.54 cm (1”) diameter smaller wafers were fabricated from each large wafer. Processing steps included thermal oxidation, lithography and etching to pattern the oxide, and deep-reactive-ion etching to thin the silicon and to define the holes in the membrane. The processing is shown schematically in Fig. 1. Thermal oxide was used to create a mask resistant to deep-reactive-ion etching, as photoresists can be problematic when used to delineate fine features during the extended etch times required for high-aspect-ratio etching. Thinning was also required for the smallest hole sizes in order to ensure that holes completely penetrated through the thickness of the silicon. Processing the wafer from two sides made it possible to perform the definition and etching of the membrane holes separately from the step used to define and etch the thinned region of the wafer. Separate definition of holes and thinned regions allowed for the fabrication of through-thickness holes with dimensions approaching 1 μm while maintaining structures that could be handled, albeit, gently.

**Fig. 2:**
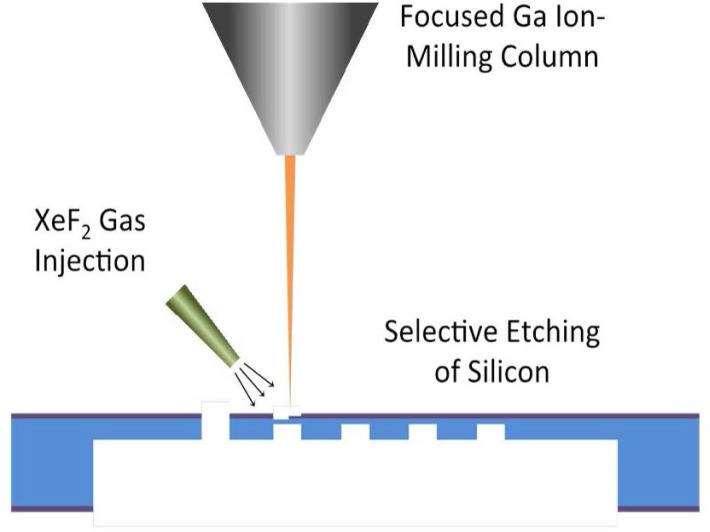
Hybrid fabrication approach using microfabrication techniques for bulk silicon removal and XeF_2_ gas assisted FIB milling for selective silicon milling.

While microfabrication excels at high-throughput and high-accuracy production of membrane structures, it is not optimal for development work because of the time and cost associated with producing the number of masks needed to accommodate various membrane designs. In this work, a hybrid approach combining the use of microfabrication and focused ion beam (FIB) milling was adopted. FIB milling is a technique optimized for precise removal of small amounts of material without the use of masks. The combined use of gallium-ion milling with the introduction of XeF_2_ gas increased silicon-etch rates to levels high enough to remove the remaining material needed for this procedure. A schematic of the approach is shown in Fig. 2, which entails performing most of the silicon etching *via* microfabrication, while performing the final hole-opening step with FIB milling.

### 2.2 Test Chamber

**Fig. 3:**
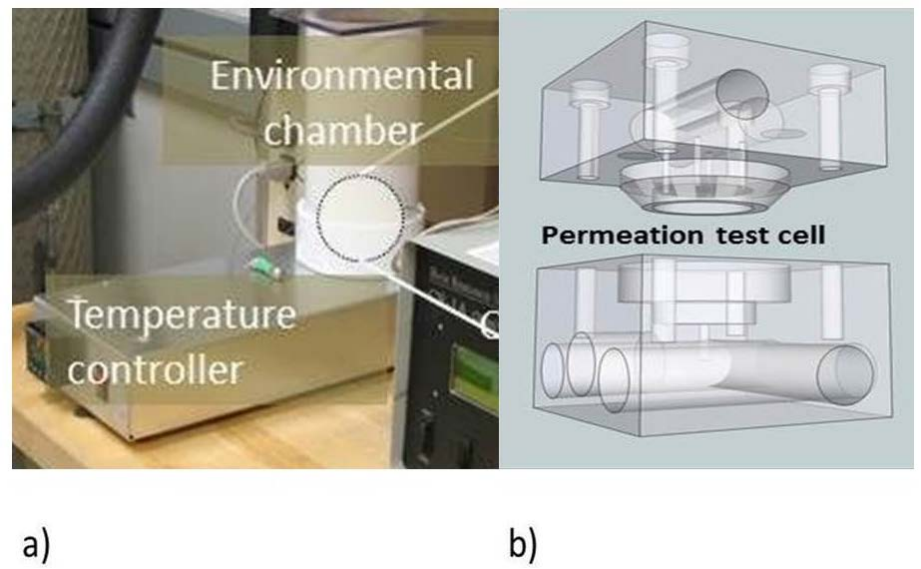
Test cell and environmental chamber. a) Temperature controller and environmental chamber. The controller is built in-house. The chamber is a plastic tube with insulation lining the walls and a cap on top to restrict air movement. b) The test cell separates to allow 25 mm diameter specimens to be mounted. Liquid water inserted on one side of the specimen permeates to the other side, where it is collected by flowing high purity nitrogen gas.

The test chamber used for both the hygrometer and mass loss measurements is shown in Fig. 3. Figure 3a shows the thermal chamber that houses the specimen test cell. The chamber consists of a section of PVC piping lined with a flexible heating pad. The tip of a T-type thermocouple was placed in direct thermal contact with the stainless steel body of the test cell. A software-controlled proportional-integral-derivative (PID) controller^1^ was used to drive a solid-state relay, providing power to the heating pad. Temperature data were collected every 2 minutes throughout testing. The ends of the chamber were closed to prevent air circulation. The permeation test cell (Fig. 3b) was a two-part, stainless steel structure designed to hold 25 mm diameter circular specimens up to a few hundreds of micrometers thick. The specimen was mounted on an annular ridge above a chamber of about 1 mm^3^ and sealed with an ethylene propylene (EPDM) O-ring. Water injected into the 1 mm^3^ space behind the specimen permeated through to the other face and was picked up by flowing ultra-high purity N_2_ gas passed across the specimen. The N_2_ flow was controlled by a commercial mass flow controller^2^ which was set to pass 0.500 SLPM (5.00 × 10^−4^ m^3^/min) at an internally controlled 1 atmosphere pressure and 25 °C.

The ultra-high purity (UHP) nitrogen gas used for testing^3^ was specified by the supplier to contain less than 1 μL/L water vapor. To further reduce the water vapor concentration, the sweep gas was directed sequentially through two glass traps containing 13X and 4 Å molecular sieve.^4^ Baseline water content in the dried sweep gas varied among gas cylinders; concentrations ranged between 200 parts-per-billion by volume (nL/L) and 500 nL/L, which corresponded to a vapor pressure range of ≈17 mPa to ≈44 mPa, or a frost point range from about −90 °C to −80 °C at the average system pressure of 87.02 kPa ± 0.24 kPa. Because the baseline was determined by the source gas, the residual water concentration was independent of flow rate once the system dried down. The N_2_ flowed to a hygrometer, in which the water content of the gas was determined. The N_2_ lines to the test cell could be disconnected and the ports to the cell capped. This allowed the test cell to be removed and weighed periodically and, subsequently, to be reintroduced into the gas flow stream.

### 2.3 Water Flow Measurements

Two methods were used to monitor water vapor passage through the specimens: chilled mirror hygrometer and mass loss.

#### 2.3.1 Hygrometer

The chilled mirror hygrometer was a commercial instrument^5^ that operated according to the basic definition of frost point (or dew point) by maintaining an even layer of frost on the chilled mirror in equilibrium with the moisture in the sample gas stream. The hygrometer used for this study was chilled with liquid nitrogen, which allowed for a lower detection limit of 10 nL/L as reported by the manufacturer. In practice, residual water vapor in the sweep gas was the factor limiting detectability, not the intrinsic sensitivity of the instrument.

The hygrometer used a photodiode pair to emit light continuously onto, and measure the corresponding reflectance from, the chilled mirror. Condensed water or frost on the mirror reduced this reflectance and, by active control of the mirror temperature *via* the PID controller loop, a condensed layer of constant thickness was maintained. The hygrometer’s PID balance data, internal absolute pressure, mirror temperature, and relative concentration were collected every 1.6 s and stored on the PC.

The frost layer on the mirror was sensitive to the process of refilling the built-in cryostat with liquid nitrogen. Addition of liquid nitrogen generated turbulence that over-cooled the mirror, causing too much frost to accumulate and resulting in the signal spikes apparent in following data plots. Subsequent to such spikes, the system had to reestablish equilibrium, which consumed a substantial amount of the interval between refills. The presence of the spikes complicated data interpretation; the procedure used to analyze the data is described in the Data Reduction section.

#### 2.3.2 Mass Loss

Mass loss measurements were conducted using a commercial balance^6^ with a maximum load capability of 205 g and resolution of 0.01 mg. Mass measurements were typically made once a day and consisted of disconnecting the test cell from the N_2_ line, closing the gas input and output lines with caps, and placing the entire test cell on the scale. Five measurements were made of the test cell each time the cell was disconnected for mass determination. Before each of the five measurements, a 200 g calibration standard was weighed. After the mass of the test cell was determined, the cell was replaced into the test chamber, the N_2_ gas was reconnected, and the permeation measurement was continued.

### 2.4 Microscopy

An electron scanning electron microscope^7^ (SEM) was used to image the Si wafers. Images were captured using a commercial software program^8^ and areas were calculated using a freeware program.^9^

## 3. Results and Discussion

Five Si wafers were fabricated for this work. A large portion of the work was concerned with assessing the reproducibility of the permeation results for a given specimen and the stability of the wafers over time. Table 1 lists the specimens, hole areas, and dates of measurements.

### 3.1 Hygrometer Results

#### 3.1.1 Typical Raw Data

**Fig. 4:**
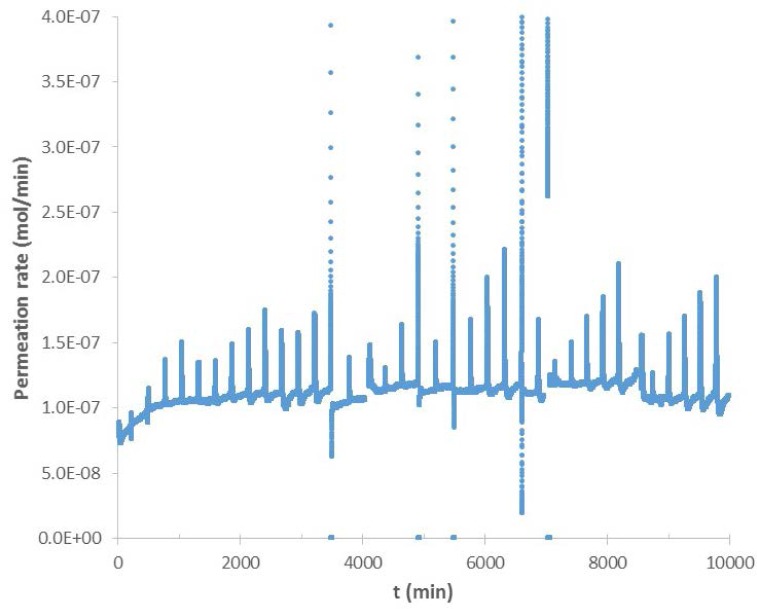
Raw hygrometer data for water permeation through Si 2 (start date 4/5/2013) showing large artifacts at each liquid nitrogen refill.

As mentioned above, data from the hygrometer showed large deviations whenever the cryostat was filled with liquid nitrogen. Figure 4 is an example of the raw data obtained from the hygrometer. Large excursions that occur about every 2.5 hours shown in Fig. 4 are typical of all of the raw hygrometer data when the liquid nitrogen is added to the cryostat; the resulting turbulence cooled the cold finger causing an increase in water deposition upon the mirror. The PID controller interpreted the reduced reflectivity from the mirror as increased moisture content in the nitrogen carrier gas. Consequently, the raw data show an increase in moisture content. The control loop increased the temperature of the mirror to maintain a constant reflectivity and drive off moisture from the mirror. However, since the water content in the gas had not increased, the heating of the mirror which drove off some of the water resulted in too high a reflectivity as the turbulence ended. At this point, the controller dropped the temperature of the mirror in order to achieve the desired reflectivity. However, because the water content in the gas had remained constant throughout this process and the apparent change in water content was strictly an artifact due to the nitrogen turbulence, the raw data show a drop in water concentration that is as unreal as the preceding increase shown in the raw data. To analyze the data from the hygrometer it was necessary to develop a procedure for removing the artifacts due to the liquid nitrogen refills. Whatever process was used to delete the artifacts had to be shown not to affect the real data. Two steps were used to achieve this. The first step was to demonstrate both that the data obtained upon one specimen is reproducible for that specimen and that removal of the LN-induced artifacts did not alter the actual permeation data. The second step was to compare hygrometer data with mass loss data. Both of these approaches will be discussed below.

#### 3.1.2 Hygrometer Model

Hygrometer results were analyzed according to the method outlined by Buck [5]. The saturated partial pressure of water in N_2_ over ice, *e*_i_*^’^*, is *f*_i_e_i_ where *e*_i_ is the saturated pressure of water over ice in the absence of other gases, e.g., air, N_2_, and *f_i_* is the enhancement factor, defined as *e*_i_*^’^/e*_i_. The expression used in this work for *e*_i_ is given by *e*_i3_ in Table 2 and Eq. (1), both in the reference:

$$e_{i3}=6.1115\exp\left(\frac{\left(23.036-\frac{T}{333.7}\right)T}{T+279.82}\right).$$ (1)

The enhancement factor is given by f_i4_ in Table 3 and Eq. (2) in the same reference:

$$f_{i4}=1+2.2\;x\;10^{-4}+P_{T}\left(3.83\;x\;10^{-6}+6.4\;x\;10^{-10}T\right)$$ (2)

where T is the temperature in K, P_T_ is the total pressure, and both P_T_ and e_i3_ are in mbar. For convenience, since the water partial pressure values measured in this work are on the order of 0.001 mbar, the pressures reported here are all converted to Pa (1 mbar = 100 Pa). Both P_T_ and T are measured in the hygrometer test chamber. The maximum fractional uncertainty,* Δe*_i3_/*e*_i3_’ < 0.0014, which is considerably less than the experimental uncertainty and, henceforth, will be ignored.

Since the water partial pressure on the dry side of the wafers was ≈ 0.1 Pa, the water is highly dispersed and can be treated as an ideal gas. Consequently, both water and N_2_ behaviors can be described by the ideal gas law:

PV=nRT (3)

in which the parameters are, in order, pressure, volume, number of moles, gas constant, and temperature. Consequently,

$$\frac{P_{w}}{P_{N}}=\frac{n_{w}}{n_{N}}=\frac{\eta_{w}}{\eta_{N}}$$ (4)

where η_i_ is the steady state flow rate of water (i = w) or N_2_ (i = N) in moles/min. P_T_, rather than P_N_, was measured in the test chamber. However,

$$P_{T}=P_{N}+P_{w}\Rightarrow P_{N}=P_{T}-P_{w}.$$ (5)

From the two expressions above, the water vapor permeation is

$$\eta_{w}≅\frac{P_{w}}{P_{N}}\eta_{N},$$ (6)

measured in mol/min. All of the parameters on the right hand side of the expression above are determined experimentally. *P_w_* and *P_T_* are measured in the hygrometer. *η_N_* is determined by the mass flow controller for the dry N_2_ gas. The flow of the N_2_, in SLPM at 25 °C and one atmosphere, was converted to mol/min by multiplying 0.500 SLPM by the density of N_2_, 1.2498 g/l and dividing by the molecular weight of N_2_. The precision of the mass flow controller was such that, evaluated over several randomly selected days, no deviation from 0.500 SLPM was observed.

In the hygrometer, e_i3_^’^ is the same as P_w_. This is not true in the permeation cell, where, at 37 °C, the partial pressure of the water is not saturated. Therefore, both e_i3_’ and P_w_ notations are maintained above. However, because there is no external source or sink for water, η_w_/η_N_ is the same at all temperatures.

#### 3.1.3 Data Reduction

**Fig. 5:**
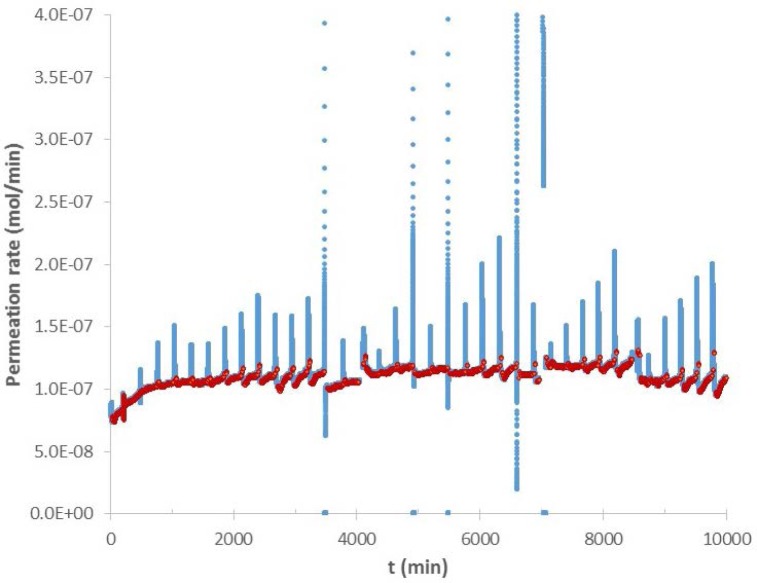
Same raw data as shown in Fig. 4. Overlay is data set modified as described in the text. The modified data set follows the contours of the raw data set but eliminates most but not quite all, of the artifacts generated by the liquid nitrogen refills.

To eliminate artifacts due to liquid nitrogen refills, the raw data were smoothed using a fast Fourier transform (FFT). The FFT calculations were made both on 100 point segments and 500 point segments to make certain artifacts were not being generated by the segment size. Once the data were smoothed, a first order derivative was taken of the data and all values of the derivative above or below threshold points were eliminated. The threshold points were chosen to eliminate most of the large excursions in the derivative. To make sure that this process was not generating artifacts in the data, the reduced data sets were plotted on top of the raw data and were seen to preserve the trends in the raw data. As an extra precaution, the threshold for the derivative was chosen so that not quite all of the excursions due to the liquid nitrogen refill were eliminated. Therefore the data reduction process eliminated most of the artifacts due to the refill but did not eliminate so much of the data that new artifacts were generated. Figure 5 is an example of raw data with the reduced data set plotted on top of it.

The hygrometer data shown hereafter fall into two categories: dry down and wet. The term dry down describes the process by which residual water from the atmosphere, the silicon wafers, the test chamber, or the experimental nitrogen gas lines was removed from the apparatus. Typically, the data were initially high, with water vapor flow rates of ≈2 × 10^−8^ mol/min, and fell over time to values on the order of 3 × 10^−9^ mol/min to 9 × 10^−9^ mol/min. This value varied with N_2_ gas cylinder, since the water content of the high purity nitrogen cylinders was not specifically controlled and varied from about 200 ppb to 900 ppb. Once steady-state had been achieved in the dry-down process, water was added to the test chamber and the hygrometer data showed an immediate increase in water permeation that leveled off between 3.5 × 10^−8^ mol/min and 1.5 × 10^−7^ mol/min, depending upon the total hole area in the specific silicon wafer.

**Fig. 6:**
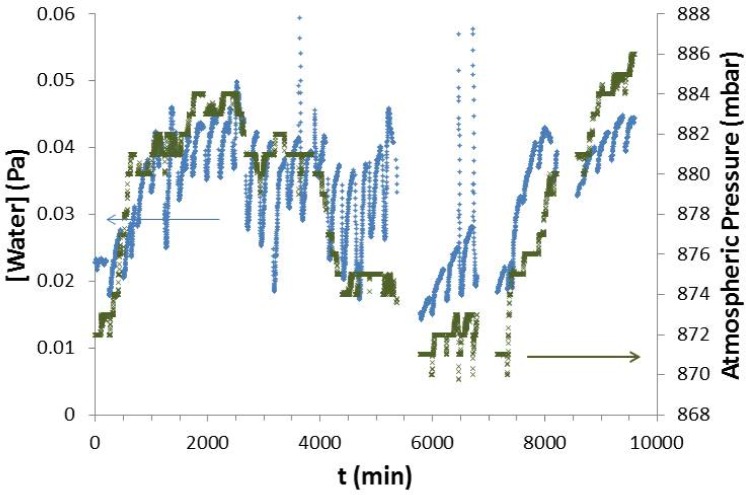
Plot of one of the dry down runs for Si 3 (blue) overlaid with atmospheric data for Boulder, CO over the same time period. Variations can be seen easily in dry down data but are difficult to observe in the water permeation data.

As described above, addition of liquid nitrogen to the system generated substantial noise. However, in the dry down process, other sources of noise appeared. One of these sources was change in atmosphere barometric pressure. Figure 6 shows data from the hygrometer on one vertical axis and the barometric pressure on the other vertical axis both plotted as a function of time. It can clearly be seen that changes in barometric pressure cause changes in the steady-state value of the dry system. However, these variations are small enough to be lost in the noise in the steady-state system with water added, and were ignored. At least one other source of noise exists, but efforts to track it down have not been successful. The noise from that source appears and disappears but the noise level is comparable to that due to barometric pressure changes and, therefore, it does not significantly affect measurements when water is in the system.

#### 3.1.4 Permeation Analysis

The transient behavior of the data from the hygrometer, both in dry down and in wet measurements, can be fitted empirically by a power law expression within experimental uncertainty. Such an expression levels off as time approaches infinity. For this work, each of the fitted curves was evaluated at 10,000 minutes.

#### 3.1.5 Reproducibility and Stability

Table 2 summarizes the measured water vapor permeation for the four silicon wafers. The table includes the total surface area of the holes, the dates at which measurements were made, and the permeation values and standard deviations at 10,000 minutes. For example, specimen Si 3, with a hole area 2213 μm^2^, was measured twice, starting 9/13/2012 and 2/4/2013. The italicized rows in Table 2 showed anomalously high rates for the last test of specimens Si 2 and Si3. Upon removal from the permeation system, it was found that both specimens had cracked. Implications of crack formation in the wafers will be discussed below.

### 3.2 Mass Loss Results

#### 3.2.1 Typical Raw Data

**Fig. 7:**
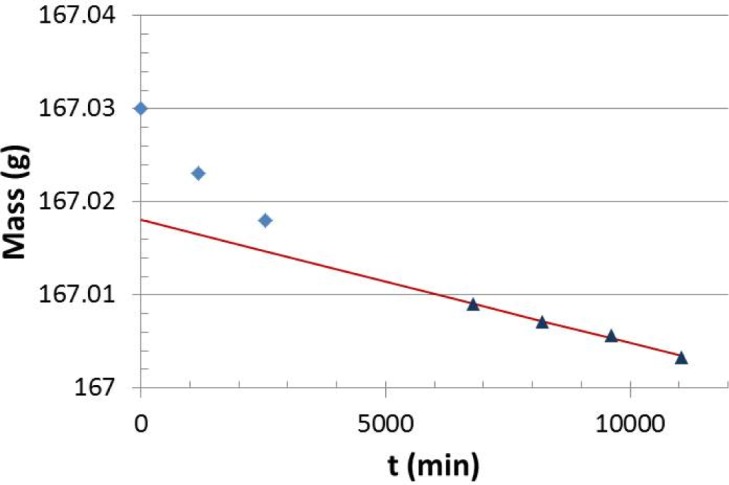
Typical plot of test cell mass as a function of time. These data were from one of the Si 4 runs.

Mass loss data were obtained for specimens Si 3 and Si 4. Specimen Si 2 cracked before mass loss measurements were made. Figure 7 is a plot typical of that observed for the mass loss data that were obtained. This plot was made for specimen Si 4, which had a total surface area of 373 μm^2^. During the initial part of such plots the mass decreased relatively rapidly at short times and then reached the steady-state mass loss rate value at longer times. A plot of the linear regression between time and mass (Fig. 7) was made for the steady-state portion of the curve for each specimen.

#### 3.2.2 Hole Surface Area Results

Table 3 summarizes the steady state mass loss (mol/min) data. Mass loss data were obtained for 3 runs on Si 4 and one run on Si 3. The data on Si 4, which were the most demanding to obtain due to the small hole surface area, resulting in an average water permeation of 6.78 × 10^−8^ mol/min with a standard deviation of 9.5 × 10^−9^ mol/min (14 %).

### 3.3 Comparison of Hygrometer and Mass Loss Experiments

#### 3.3.1 Models

**Fig. 8:**
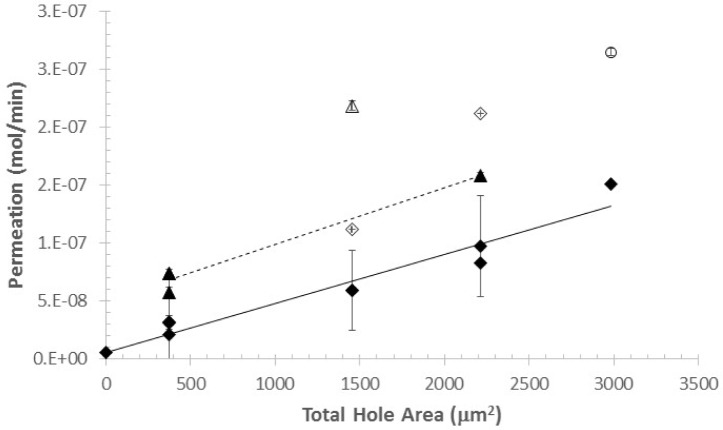
Summary plot of water permeation (mol/min) as a function of silicon hole area for both un-cracked (solid symbols) and cracked (open symbols) wafers. Lines are linear fits between permeation and hole area for hygrometer (solid) and mass loss (dashed) measurements. The hollow circular point corresponds to mass loss due to what appeared to be a point-load-induced lateral crack on the underside edge of the specimen.

Figure 8 and Table 4 summarize the hygrometer and mass loss data for the silicon wafers. Figure 8 is a plot of the water vapor permeation as a function of the total hole area in the wafer. The solid and open diamonds reflect hygrometer results for the un-cracked and cracked wafers, respectively. Similarly, the solid and open triangles reflect the mass loss data for the un-cracked and cracked wafers. In all cases, the error bars reflecting standard deviation are included in each of the data points. The two lines are the linear regression of the permeation onto the hole areas. Table 4 provides the slopes, intercepts, and standard errors for the two cases. Both the slopes of the lines and the intercepts are important for evaluating the suitability of silicon wafers with micro-machined holes as standards for low permeation level water permeation standards.

The dependence of the measured water permeation on total hole area was observed to be linear and the slopes of the two lines, hygrometer and mass loss, were indistinguishable. Therefore, the change in permeation rate with total hole area is well defined and predictable. In addition, the agreement between the slope determined by the mass loss measurements with the slope determined by the hygrometer measurements indicates that the procedure used to remove the LN refill artifacts did not generate new artifacts that would affect the change in permeation rate evaluation as hole area changes. The relative uncertainty in the slope of the hygrometer data was 5.9 %, which provides a lower limit to the accuracy of the silicon wafers being used to calibrate for changes in water permeation.

The intercepts provide an estimate of the uncertainty of individual measurements of the water pressure. The intercept for the hygrometer measurements was found to be 5.06 × 10^−9^ mol/min ± 5.2 × 10^−9^ mol/min (Table 4); the standard error for the intercept overlapping zero. The mass loss data has a larger intercept offset than the data from the hygrometer, 5 × 10^−8^ mol/min. The design of the permeation cell includes an O-ring that is clamped between the specimen and the dry N_2_ side of the cell. There is no O-ring between the specimen and the side of the test cell containing the water reservoir. Our interpretation for the constant offset of the mass loss data from the hygrometer data is that water vapor is escaping from sides of the permeation cell, between the test cell wall and the test specimen. An attempt was made to place an O-ring on the reservoir side of the specimen. The measurement was made with a Si blank (i.e., a wafer containing no holes) as a specimen. The extra thickness due to the addition of the O-ring resulted in a significantly larger mass loss, although hygrometer measurements were consistent with baseline water content of the N_2_ gas. All of these results are consistent with water vapor escaping out the sides of the test cell.

From the data in Fig. 8 and Table 4, the following conclusions can be drawn.

- The response of water pressure measuring systems to total hole area is linear over the range of 370 μm^2^ to 2200 μm^2^.
- Hygrometer measurements show that relative changes in permeation can be evaluated with the silicon wafers to approximately 6 %.

Another feature of the silicon wafers is shown in Fig. 8 for both the hygrometer and mass loss data. Cracks in the wafers (e.g., Si 2 and Si 3) result in discrete increases in measured water pressure that are significantly outside the normal measurement error bars. This is in contrast to polymer coatings, which can be expected to exhibit a continuous change in water pressure as the material degrades over time. The difference results from the fact that Si, with a SiO_2_ surface coating is both chemically stable and almost completely brittle. Therefore, it would not be expected to degrade chemically over time and it would not exhibit plastic deformation that would change the hole areas. Consequently, any damage to the surface of the Si wafers would be expected either to have no effect on permeation rate, e.g., surface chips, or to result in easily detected, large, discrete changes in permeation, e.g., cracks (see Fig. 8). On the negative side, because Si is brittle and the thickness of the wafers in the vicinity of the holes is on the order of micrometers, the wafers are quite fragile; small shear loading will result in crack formation, destroying the calibration of the wafer.

## 4. Summary

Five Si wafers (Si 1, no holes; Si 2, 1452 μm^2^ total hole area; Si 3, 2213 μm^2^ total hole area; Si 4, 373 μm^2^ total hole area; and SiF1, 2984 μm^2^ total hole area) were evaluated to assess the use of Si wafers as permeation standards for water. Measurements were made with both frost point hygrometer and mass loss techniques. All of the mass loss measurements were made in parallel with the hygrometer, giving independent measurements on the same wafer, under the same conditions, over the same time period.

Results indicated that micro-machined holes in Si wafers perform well as permeation standards:

- The data showed a well-defined linear relationship between total hole area and subsequent steady-state permeation rate. Differences in permeation rate of ≈ 10^−8^ mol/min were easily detected and related to total hole area.
- Permeation through the Si wafers was demonstrated to be quite stable over time, unlike polymer permeation calibration standards currently in use.
- Micro-machining of Si is a well-established technology, so calibration standards of desired permeation can be manufactured accurately and reproducibly.
- Damage to the wafers (cracks) results in large, discontinuous changes in permeation and, consequently, is easily detected.

However, thinned Si wafers were very fragile and were easily cracked. Therefore, the finished wafers require that very delicate handling, cleaning, and storage procedures be developed.

## Figures and Tables

**Table 1:** Summary of Si wafers, including start and end dates of measurements. Dry Down is the period between the time the wafer is placed into the test chamber and the time that the measured water content is reduced to the equilibrium value define by the water content of the nitrogen carrier gas. Time from First Run is measured from the start of the Dry Down procedure. (Italics indicate runs for which the Si wafer was subsequently determined to have been cracked.)

		Dry down		Permeation				
				
Sample	Description	Start date	End date	Start date	End date	Mass-loss measured?	Hole area (μm^2^)	Time from First Run (Days)
Si wafer 1	blank	3/31/12	4/5/12	4/6/12	4/9/12	No		0
Si wafer 1	blank	7/26/12	8/15/12	NA	NA	No	0	117
								
Si wafer 2	5 holes	5/13/12	5/15/12	5/16/12	5/22/12	No		0
Si wafer 2	5 holes	7/6/12	7/12/12	7/13/12	7/16/12	No	1452	85
*Si wafer 2*	*5 holes*	*3/29/13*	*4/5/13*	*4/5/13*	*4/12/13*	*Yes*		*325*
								
Si wafer 3	5 holes	8/27/12	9/13/12	9/13/12	9/20/12	No		0
Si wafer 3	5 holes	2/1/13	2/4/13	2/4/13	2/13/13	Yes	2213	158
Si wafer 3	5 holes	2/15/13	2/20/13	NA	NA	No		172
*Si wafer 3*	*5 holes*	*3/1/13*	*3/4/13*	*3/4/13*	*3/5/13*	*Yes*		*186*
								
Si wafer 4	2 holes	2/20/13	2/21/13	2/21/13	3/1/13	Yes		0
Si wafer 4	2 holes	3/5/13	3/6/13	3/6/13	3/29/13	Yes	373	14
								
Si wafer F1	5 holes	5/19/15	5/28/15	5/28/15	6/5/15	No	2944	0

**Table 2:** Summary of the water permeation (mol/min) for the five wafers, measured from the hygrometer. A description of the number of holes in each specimen, the total hole areas, and the dates of the runs are included. The values and standard deviation values are provided for 10,000 minutes after the beginning of each experiment. The values were obtained by fitting the data, after removal of LN-generated artifacts (see text) and subtraction of the baseline determined from the Dry-Down data. Italicized rows correspond to data taken from cracked samples.

Wafer	Description	Date	10,000 minutes	Std dev	Hole Area (μm^2^)
Si 1	blank	3/31/2012	−1.19 × 10^−10^	5.1 × 10^−10^	0
Si 1	blank	7/26/2012	5.26 × 10^−^9	9.3 × 10^−10^	0

Si 2	5 holes	5/16/2012	5.91 × 10^−8^	3.49 × 10^−8^	1452
Si 2	5 holes	7/6/2012	5.91 × 10^−8^	3.4 × 10^−11^	1452
*Si 2*	*5 holes*	*3/29/2013*	*1.11 × 10^−7^*	*1.2* × *10^−11^*	*1452*

Si 3	5 holes	9/13/2012	8.31 × 10^−8^	4.0 × 10^−11^	2213
Si 3	5 holes	2/4/2013	9.65 × 10^−8^	4.4 × 10^−8^	2213

*Si 3*	*5 holes*	*3/1/2013*	*2.12* × *10^−7^*	*8.6* × *10^−11^*	*2213*
Si 4	2 holes	2/21/2013	2.12 × 10^−8^	1.5 × 10^−11^	373
Si 4	2 holes	3/6/2013	3.14 × 10^−8^	5.9 × 10^−9^	373
Si 4	2 holes	3/15/2013	3.05 × 10^−8^	4.3 × 10^−8^	373

Si F1	1 hole	5/28/2015	1.51 × 10^−7^	4.2 × 10^−11^	2984

**Table 3:** Summary of mass loss results. Due to crack formation in two of the Si wafers, only three runs for Si 4 and one run for Si 3 were obtained in the undamaged specimens.

Specimen	Area (μm^2^)	Permeation (mol/min)	Std dev (mol/min)	Date
Si 4	375	5.68 × 10^−8^	4.6 × 10*^−^*^9^	2/13/2013
Si 4	375	7.33 × 10^−8^	4.8 × 10^−9^	3/6/2013
Si 4	375	7.33 × 10^−8^	3.4 × 10^−^9	3/15/2013

Si 3	2213	1.58 × 10^−7^	2.9 × 10^−9^	2/4/2013

**Table 4:** Summary of the linear fits shown in Fig. 8

	Slope (mol/min/cm^2^)	Std error	Intercept (mol/min)	Std error
**Hygrometer**	4.26 × 10^−11^	3.4 × 10^−12^	5.06 × 10^−9^	5.2 × 10^−9^
**Mass Loss**	4.89 × 10^−11^	6.0 × 10^−12^	4.96 × 10^−8^	6.9 × 10^−9^

## Footnotes

1 Omega CNi16D43-EI, Omega Engineering, Inc., Stamford, CT. Company information is provided for experimental completeness and does not imply an endorsement by the National Institute of Standards and Technology.

2 MC-1SLPM-D, Alicat Scientific, Inc., 7641 N. Business Park Dr., Tucson, AZ 85743.

3 Specimens Si 1 – Si 4: UHP plus nitrogen #LW411P, Matheson Tri-Gas, Inc. division of Taiyo Toyo Sanso Corporation, Tokyo, Japan. Specimen Si F1: Research Nitrogen 6. #121 1213567, General Air, Boulder CO, USA.

4 Agilent LGMT-2-HP, Agilent Technologies, Inc., Santa Clara, CA.

5 Buck CR-1A, Buck Research Instruments, LLC, Boulder, CO.

6 Mettler Instrument Corp., Mettler AT201, Mettler-Toledo, LLC, 1900 Polaris Parkway, Columbus, OH 43240.

7 JSM-6100 Scanning Microscope, JEOL USA, Inc., 11 Dearborn Road, Peabody, MA 01960.

8 Spirit 1.07.02, Princeton Gamma-Tech, RIID Repairs Thermo Fisher Scientific 312 Miami St, West Columbia, SC 29170 USA.

9 ImageJ 1.44p, http://rsbweb.nih.gov/ij/.
